# Supplementary material for: Causal Relationship between Aging and Anorexia Nervosa: A White-Matter-Microstructure-Mediated Mendelian Randomization Analysis
Source: Biomedicines. 2024 Aug 16;12(8):1874. doi: 10.3390/biomedicines12081874 (PMC11351342; doi:10.3390/biomedicines12081874)
Supplement: Supplementary file 1 [file biomedicines-12-01874-s001.zip › Supplementary Figure/Figure S4/Singe SNP plot of MR analysis for TL to ubm-b-1763.pdf]

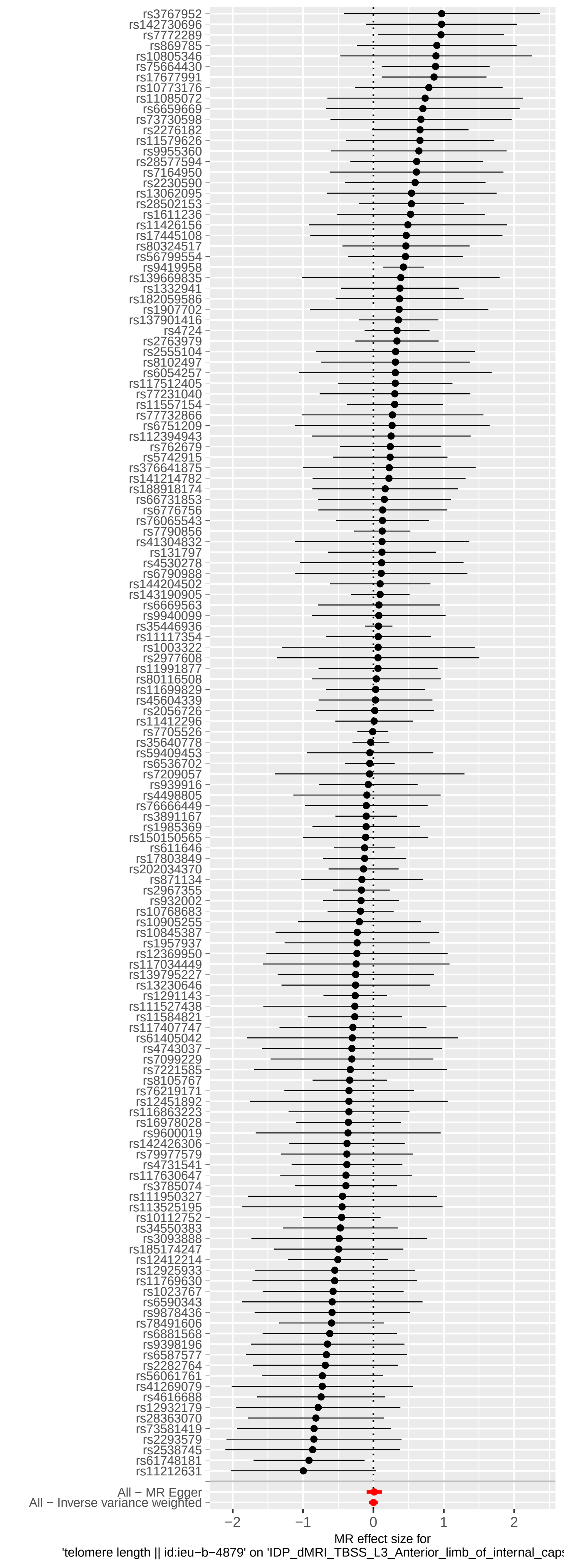

rs3767952
rs142730696
rs7772289
rs869785
rs10805346
rs75664430
rs17677991
rs10773176
rs11085072
rs6659669
rs73730598
rs2276182
rs11579626
rs9955360
rs28577594
rs7164950
rs2230590
rs13062095
rs28502153
rs1611236
rs11426156
rs17445108
rs80324517
rs56799554
rs9419958
rs139669835
rs1332941
rs182059586
rs1907702
rs137901416
rs4724
rs2763979
rs2555104
rs8102497
rs6054257
rs117512405
rs77231040
rs11557154
rs77732866
rs6751209
rs112394943
rs762679
rs5742915
rs376641875
rs141214782
rs188918174
rs66731853
rs6776756
rs76065543
rs7790856
rs41304832
rs131797
rs4530278
rs6790988
rs144204502
rs143190905
rs6669563
rs9940099
rs35446936
rs11117354
rs1003322
rs2977608
rs11991877
rs80116508
rs11699829
rs45604339
rs2056726
rs11412296
rs7705526
rs35640778
rs59409453
rs6536702
rs7209057
rs939916
rs4498805
rs76666449
rs3891167
rs1985369
rs150150565
rs611646
rs17803849
rs202034370
rs871134
rs2967355
rs932002
rs10768683
rs10905255
rs10845387
rs1957937
rs12369950
rs117034449
rs139795227
rs13230646
rs1291143
rs111527438
rs11584821
rs117407747
rs61405042
rs4743037
rs7099229
rs7221585
rs8105767
rs76219171
rs12451892
rs116863223
rs16978028
rs9600019
rs142426306
rs79977579
rs4731541
rs117630647
rs3785074
rs111950327
rs113525195
rs10112752
rs34550383
rs3093888
rs185174247
rs12412214
rs12925933
rs11769630
rs1023767
rs6590343
rs9878436
rs78491606
rs6881568
rs9398196
rs6587577
rs2282764
rs56061761
rs41269079
rs4616688
rs12932179
rs28363070
rs73581419
rs2293579
rs2538745
rs61748181
rs11212631
All − MR Egger
All − Inverse variance weighted
−2
−1
0
1
2
MR effect size for
'telomere length || id:ieu−b−4879' on 'IDP_dMRI_TBSS_L3_Anterior_limb_of_internal_caps
